# Missing Dosages and Neuroleptic Usage May Prolong Length of Stay in Hospitalized Parkinson's Disease Patients

**DOI:** 10.1371/journal.pone.0124356

**Published:** 2015-04-17

**Authors:** Daniel Martinez-Ramirez, Juan C. Giugni, Christopher S. Little, John P. Chapman, Bilal Ahmed, Erin Monari, Aparna Wagle Shukla, Christopher W. Hess, Michael S. Okun

**Affiliations:** 1 Department of Neurology, University of Florida College of Medicine, Center for Movement Disorders and Neurorestoration, Gainesville, Florida, United States of America; 2 Department of Neurosurgery, University of Florida College of Medicine, Center for Movement Disorders and Neurorestoration, Gainesville, Florida, United States of America; University of Pennsylvania Perelman School of Medicine, UNITED STATES

## Abstract

**Background:**

Parkinson’s disease patients are more likely to be hospitalized, have higher rates of hospital complications, and have an increased risk of deterioration during hospitalization. Length of stay is an important underlying factor for these increased risks. We aimed to investigate potential medication errors that may occur during hospitalization and its impact on length of hospital stay.

**Methods:**

A cross-sectional chart review of 339 consecutive hospital encounters from 212 PD subjects was performed. Medication errors were defined as wrong timing or omission of administration for dopaminergic drugs and administration of contraindicated dopamine blockers. An analysis of covariance was applied to examine whether these medication errors were related to increased length of hospital stays.

**Results:**

A significant effect for dopaminergic administration (p<0.01) on length of hospital stay was observed. Subjects who had delayed administration or missed at least one dose stayed longer (M=8.2 days, SD=8.9 vs. M=3.6 days SD=3.4). Contraindicated dopamine blocking agents were administered in 23% (71/339) of cases, and this was also significantly related to an increased length of stay (M=8.2 days, SD=8.9 vs. M=3.6 days SD=3.4), p<0.05. Participants who received a contraindicated dopamine blocker stayed in the hospital longer (M=7.5 days, SD=9.1) compared to those who did not (M=5.9 days, SD=6.8). Neurologists were consulted in 24.5% of encounters. Specialty consultation had no effect on the medication related errors.

**Conclusions:**

Missing dopaminergic dosages and administration of dopamine blockers occur frequently in hospitalized Parkinson’s disease patients and this may impact length of stay. These potentially modifiable factors may reduce the risk of a longer stay related to hospitalization.

## Introduction

Parkinson’s disease (PD) is a progressive neurodegenerative disorder that often leads to significant morbidity with adverse effects on the quality of life [1, 2]. Neuropsychiatric issues, urinary tract infections, swallowing related aspiration, and fall related fractures are major health-related complications that have been reported as drivers of hospitalization [3–9]. PD patients have been reported to experience a higher rate of hospitalization (approximately 30%) and re-hospitalization (50%) as compared to the general population [7]. The cumulative effect of PD-related hospitalization increases the burden of care, costs, morbidity, and mortality [6, 10].

The increasing disease burden derived from the aging population has contributed to economic and public health urgency to uncover and to implement more cost-effective interventions. Since PD patients face higher rates of hospital based complications and also have a substantially higher risk for deterioration during hospitalization [11, 12], it will be critical to identify potentially preventable risk factors and ultimately interventions that will reduce morbidity, mortality, and their associated economic burdens. Medication errors in the general population have been previously studied in various health care facilities. There has been a reported 19% overall medication administration error rate and 73% of the errors have been related to either a wrong time of administration or to omission [13]. Regarding PD, medication errors related to dopaminergics have been suggested to be an important risk factor for hospital based clinical deterioration [12]. We intended to investigate medication errors that occurred during hospital encounters and to uncover a potential relationship with length of hospital stay in a large longitudinal dataset derived from a well-characterized population of hospitalized PD patients.

## Materials and Methods

The University of Florida (UF) Institutional Review Board (IRB) approved this study. Written informed consent from study participants was obtained for research purposes under the previously IRB approved UF-INFORM database protocol. Patient records were anonymized and de-identified prior to analysis.

### Study design

A cross-sectional data/chart review of an electronic health record system (EPIC) and of a prior Institutional Review Board (IRB) approved UF-INFORM database was conducted at the UF Center for Movement Disorders and Neurorestoration (CMDNR) for the period from January 2011 to March 2013. The UF-INFORM system is a clinical-research database, which provides information on patient demographic, clinical, and functional characteristics of patients. The database currently has over 8,000 patients.

### Population studied

Diagnosis code (*International Classification of Diseases*, *Ninth Revision*, *Clinical Modification*) was used to identify subjects with PD (*ICD-9-CM* code 332.0) who had a hospital encounter for inclusion in the study. A hospital encounter was defined as an emergency room visit, being subsequently hospitalized or any direct admission to the hospital due to any cause. Subjects who were not receiving at least one dopaminergic medication or found to have hospital encounters due to an elective surgical procedure were excluded. A total of 752 hospital encounters were reviewed. There were 368 excluded because of an elective surgical procedure as the reason for hospitalization and additional 45 were excluded because patients were not receiving dopaminergics.

### Data collection

The following demographic and clinical data elements were obtained from each subject’s chart at time of the hospital encounter: age, gender, medical history, disease duration, Hoehn and Yahr (H&Y) stage, levodopa equivalent dose (LED), reason for hospitalization, length of hospitalization in days, number of hospitalizations in the stated period, in-hospital falls, whether a neurologist was consulted during the hospitalization, and the discharge disposition. In order to assess the effect of the comorbidities obtained from the medical history a Charlson index score was used [14]. The Charlson index is a scoring system for common comorbid conditions and it is weighted according to mortality risk and has been previously applied in PD studies [15]. Although dementia is included in the Charlson index, we performed a sub-analysis by grouping patients with any alteration in cognition separately based on the initial physician’s clinical note. These alterations included a mild cognitive impairment, or dementia. Reasons for hospitalization were classified into categories adapting the Emergency Severity Index (ESI) triage algorithm [16] to our known reason of hospitalization, where ESI 1 and 2 were classified as a high risk for prolonged length of stay, including falls/fractures, pulmonary causes (including pneumonia), cardiac issues/syncope, genitourinary infections, encephalopathy/drug-induced psychosis, cancer, stroke, and dementia. ESI 3–5 was classified as low risk, including general medical conditions (including weakness, pain, bleeding, or anorexia) and other medical issues (including seizures, vascular problems, or trauma), and gastrointestinal issues.

### Outcomes and measurements

The main outcome of the study was to investigate if errors in administration of medications during PD hospitalization affected the length of stay. We also intended to investigate the relationship between these medication errors and in-hospital falls.

Errors in medication administration were defined as a dose administered differently than as ordered on the patient’s medical record [13]. Two categories were used to report errors in dopaminergic administration: 1. wrong time—administration of a dose more than 60 minutes before or after the scheduled administration time; and 2. omission—failure to administer an ordered dose. We also considered a medication error in PD patients inclusive of administration of a contraindicated dopamine blocker (i.e. medications that could make the PD worse). In order to assess the dopaminergics errors, a meticulous analysis of documentation of specific aspects of medication administration during each hospital stay was performed through each subject’s chart, nursing notes, and orders were all analyzed. Wrong time of administration was obtained using the number of dosages not administered within the expected hour, number of dosages not administered within 2 hours, number of dosages not administered within 3 or more hours, and omission was obtained with the dosages completely omitted and by documenting specific reasons. Additionally, if dopaminergics were written with specific times rather than with common hospital schedules (e.g. BID, TID, QID) this was documented. Dopaminergic medications for this study were limited to levodopa and dopamine agonists. Error administration rate was reported in percentage calculated in the following manner: the number of dopaminergic errors divided by the total number of scheduled dosages during hospitalization period multiplied by 100.

In order to assess errors related to dopamine blocker administration, the electronic chart was reviewed to identify if any type of dopamine blocker agent was administered during hospitalization. Dopamine blockers were further divided in those considered contraindicated in PD, which included antipsychotics (e.g. haloperidol, risperidone, and olanzapine) and other medications such as metoclopramide, promethazine, and prochlorperazine, and in those considered indicated dopamine blocking agents in PD, such as quetiapine or clozapine [17].

### Statistical analysis

A general linear model analysis of covariance test (ANCOVA) was performed to learn whether delayed or missed doses of levodopa and/or use of a contraindicated dopamine blocker agent were related to increased length of hospital stays. Because the following variables may be expected to impact length of hospitalization, all were included as covariates in the analysis: age, Charlson Index score, reason for hospitalization (low risk versus high risk, as described in the section above), disease duration, H&Y stage, and gender. Tests of normality were run for all relevant variables. Due to positive skew greater than 1 for disease duration, length of hospital stay, number of hospital encounters, and LED, medians and interquartile ranges are reported rather than using means and standards. The remainder of the variables showed an acceptable level of normality (skewness and kurtosis values between -1 and 1). Log transformations were performed on the length of hospitalization and disease duration variables prior to all analyses and this was done due to a positive skew. Because the log transformations did not (in any case) affect the direction or significance of effects, tables and figures include raw, untransformed values to enhance interpretability. Finally, a Chi-square test was run to learn whether a consulting a neurologist had an effect on delaying or missing dopaminergic administration, in the use of a contraindicated dopamine-blocking agent, or whether a missed dopaminergic dosage was related to an in-hospital falls. A Mann-Whitney U was run to learn whether length of stay was related to cognitive status at admission, age, disease duration, or to risk of admission. Statistical analyses were performed using commercially available statistical software (SPSS, version 19.0; SPSS, Inc., Chicago, Illinois) with a priori alpha levels of ≤ 0.05.

## Results

### Study population

A total of 339 hospital encounters from 212 subjects (39.2% female, 60.8% male) were included. The cohort had a mean age 74.1 (SD = 10.1) years, a median disease duration of 4 (2–9.3) years, and mean Charlson index of 5.9 (SD = 1.4). Thirty-four percent of the patients had a clinical documentation of alteration in cognition during the initial hospital evaluation. The majority had an advanced H&Y stage (36% in stages I-III and 48.7% in stages IV-V), with a median of 1 (1–2), and the median LED was 366.7 (200–775.1) mg. Eleven patients (3.2%) suffered a fall during hospital stay. Table 1 shows the demographic and clinical characteristics of our population.

**Table 1:** Demographic and clinical characteristics of population.

	Mean (SD)	Minimum	Maximum	Median (Inter-quartile Range)
Age, y	74.1 (10.1)	34	97	75 (67–82)
Male, no. (%)	206 (60.8)	-	-	-
Charlson index	5.9 (1.4)	2	9	6 (5–7)
Disease duration, y^a^	6.4 (6.3)	0	30	4 (2–9.3)
Hoehn and Yahr	n/a	0	2	1 (1–2)
LED, mg.^a^	502.4 (487.9)	0	3285	366.68 (200–775.1)
Hospital encounters per subject^a^	2.6 (2)	1	9	2 (1–4)
Length of hospital stay, days^a^	6 (7.1)	1	63	4 (2–7)

LED: Levodopa equivalent dosage; SD: standard deviation.

^a^These variables showed a positive skew greater than 1, and thus the median and inter-quartile range should be considered rather than the mean and standard deviation.

### Characteristics of hospital encounters

The most common reasons for ER visits or admission were falls and/or fractures in 18%, pulmonary problems 15.3%, general medical conditions 15%, other medical issues 14.2%, and gastrointestinal issues 12.7%. The length of hospitalization varied between 1 and 63 days, with a mean duration of 6 (SD = 7.1) days and a median of 4 days (interquartile range 2–7). The median number of hospital encounters per subject during the period studied (27 months) was 2 (interquartile range 1–4), ranging between 1 and 9. A neurologist was consulted in about 24.5% of the hospital encounters. The most common discharge dispositions were a routine discharge to home in 31.9%, a discharge to an extended skilled nursing facility in 26.5%, and a discharge to home health care services in 18.3%. Thirteen subjects (3.8%) expired at the hospital.

### Timing of dopaminergics

**Fig 1:**
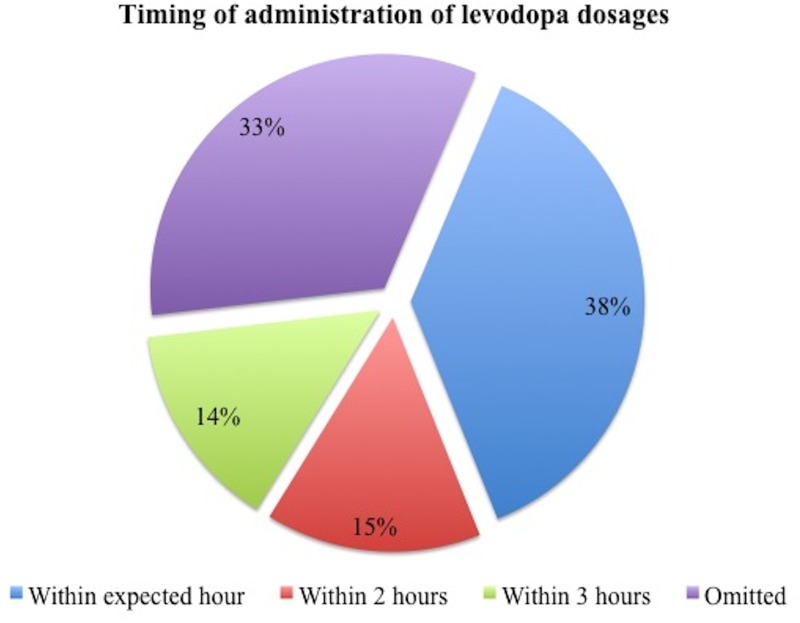
Levodopa delayed or missed dosages (n = 1507).

**Fig 2:**
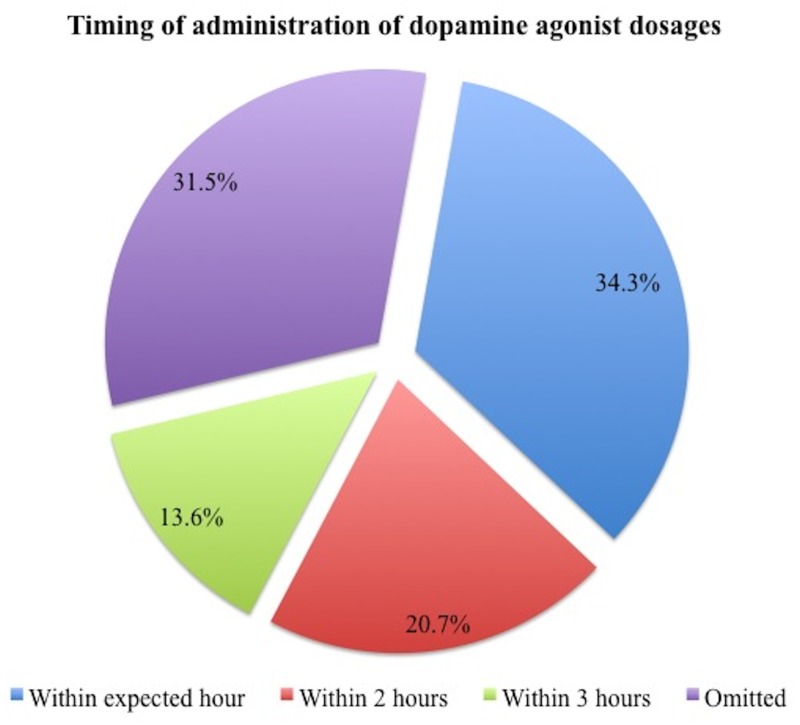
Dopamine agonists delayed or missed dosages (n = 213).

The data/chart review revealed a minority of subjects in which dopaminergic drugs were ordered with specific times for administration rather than just written as BID, TID, or QID (levodopa in 10.1% and dopamine agonists in 4.2%). When considering the total number of scheduled dosages in the cohort, 29.5% (1507/5,103) of levodopa and 24.2% (213/881) of dopamine agonists dosages were either delayed or missed. Specifically for levodopa (Fig 1), dosages were not administered within the expected hour in 11.1% (565/5,103), within 2 hours in 4.4% (225/5,103), within 3 hours in 4.2% (216/5,103), and dosage were completely omitted in 9.8% (501/5,103) of cases. For dopamine agonists (Fig 2), dosages were not administered within the expected hour in 8.3% (73/881), within 2 hours in 5% (44/881), within 3 hours in 3.3% (29/881), and were omitted in 7.6% (67/881). The reasons for dopaminergics being omitted was only reported in 41/568 dosages, being nil per os (i.e. an order to withhold oral intake) (27/41, 65.6%) and non-availability of medications (10/41, 24.3%) as the two most common reasons for omission. The relevant independent variable of interest in the ANCOVA was whether a dose of levodopa was delayed or missed during the hospital stay or not.

### Administration of dopamine blockers

Administration of any type of dopamine blocker occurred in 27.3% (90/330) of the hospital encounters for which we have data. Contraindicated dopamine blockers were used in 78.9% (71/90) of these times. Multiple or combinations of contraindicated dopamine blocking drugs were used in 47.8% of these cases. The most common prescribed dopamine blockers were haloperidol in 54.4% (49/90) and olanzapine in 36.7% (33/90). Review of the usage of dopamine blockers indicated in PD patients revealed quetiapine was used in 24.4% (22/90) and clozapine in 2.2% (2/90). The relevant independent variable of interest in the ANCOVA was whether a contraindicated dopamine blocker was administered during the hospital stay or not.

### Factors associated with length of stay

**Fig 3:**
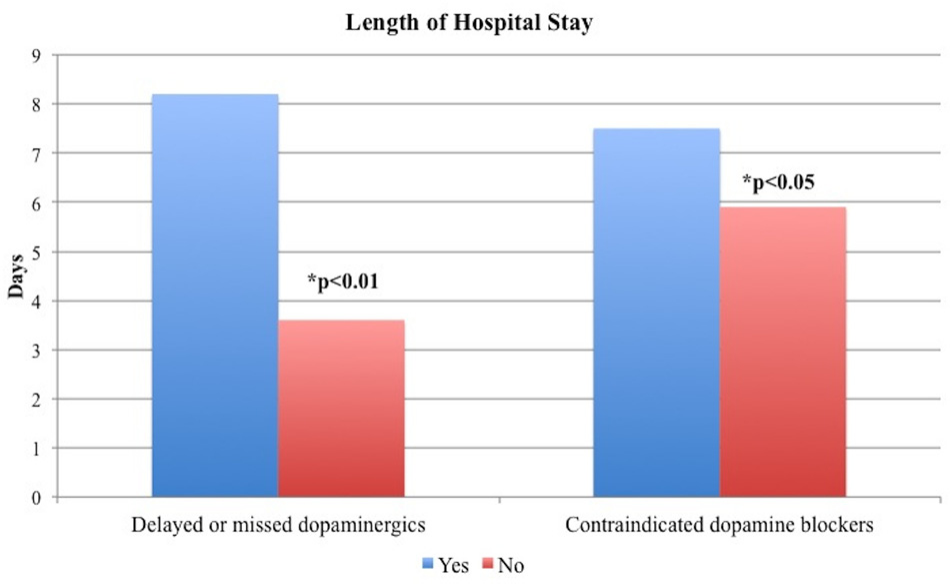
Effect of wrong administration of dopaminergic medications and neuroleptics on length of hospital stay (days).

A significant difference was observed in length of stay when grouping those with a high-risk reason of hospitalization versus those with a low-risk reason, M = 1.5 SD = 0.9 vs. M = 1.2 SD = 0.9, median 5 vs. 3 days, p = 0.005. After adjusting for confounding variables such as age, gender, Charlson index score, low or high risk hospitalization, disease duration, and H&Y stage, the ANCOVA analysis revealed a significant effect of delayed or missed doses in length of hospital stay (p<0.01). Participants who had delayed or missed at least one dose stayed in the hospital longer (M = 8.2 days, SD = 8.89, median 5 days) compared to those who did not (M = 3.6 days, SD = 3.4, median 2 days). The results also showed a significant effect of contraindicated dopamine blocker use on length of hospital stay (p<0.05). Participants who received a contraindicated dopamine blocker stayed in the hospital longer (M = 7.5 days, SD = 9.1, median 7 days) compared to those who did not, M = 5.9 days, SD = 6.8, median 4 days (Fig 3). We further analyzed how missed dopaminergic dosages could be related to in-hospital falls. A Chi square test revealed no significant difference in falls during hospitalization between those with missed dopaminergics vs. those without missing dosages, 2 vs. 4, p = 0.2. No significant differences were observed in length of stay when stratifying the population into the following groups: those with alteration in cognition versus those without alteration, M = 1.4 SD = 0.9 vs. M = 1.3 SD = 0.9, median 4 vs. 4 days, p = 0.79; those 70 years or above versus those with less than 70 years of age, M = 1.3 SD = 0.9 vs. M = 1.4 SD = 0.8, median 4 vs. 4 days, p = 0.87; and into those with 10 years or more of disease duration versus those with less duration, M = 1.3 SD = 0.9 vs. M = 1.3 SD = 0.9, median 3.5 vs. 4, p = 0.58. We also examined the distribution of patients for whom a neurologist was consulted during the hospitalization. A Pearson Chi Square test revealed that a neurologic consultation did not significantly impact whether there were delayed or missed doses of dopaminergics (p = 0.41), or whether a contraindicated dopamine blocker was given (p = 0.92).

## Discussion

We conducted a retrospective cross-sectional study of 339 hospital encounters in 212 PD patients to investigate the potential relationship between wrong administration of dopaminergics and usage of dopamine blockers with the length of hospital stay. Our results revealed that wrong administration of dopaminergics during hospitalization was associated with a detrimental effect on the length of stay, as did the use of contraindicated dopamine blockers. The following critical findings were uncovered: First, in the majority of cases, dopaminergic medications were written in simple schedules (BID, TID, QID) instead of exact and individualized time schedules. Second, dopaminergic medications were frequently not administered on time or alternatively were completely omitted. Third, the main reason for medication omission was the non-availability of medications from hospital pharmacies. Finally, dopamine blockers were frequently used in PD patients. We were surprised to find that though neurologists were infrequently consulted, this did not impact errors in medication administration.

It is well known that a suboptimal adherence to PD medications places patients at higher risk of worsening of symptoms [18, 19], and this issue highlights the importance of administering dopaminergic medications on time every time in hospitalized patients. Unfortunately, PD patients are frequently subjected to suboptimal and inexact medication schedules during hospitalization [11, 20]. We found that 29.5% of levodopa and 24.2% of dopamine agonists’ dosages were either delayed or missed. Erroneous administration of medications is an important risk factor for hospital based deterioration [12]. Additionally in non-PD populations, the use of dopamine-blocking agents [17] extends the length of the hospital stay, increases costs and morbidity, and hastens mortality [21]. However, these issues were not previously explored in a large cohort of PD patients.

Including the present study, four retrospective peer-reviewed studies explored some of these PD related hospitalization issues (see Table 2).

**Table 2:** Comparison of studies exploring medication management in the hospitalized Parkinson’s disease patient.

	Magdalinou et al. (2007)	Derry et al. (2010)	Hou et al. (2012)	*Present study* (2014)
Study design	Retrospective	Retrospective	Retrospective	Retrospective
Study period	14 months	18 months	24 months	27 months
Admissions/patients	-/35	59/54	-/89	339/212
Admission department	ER	Surgery	Hospital	ER or Hospital
Medications written with specific times	-	-	23.6%	Levodopa 10.1%; dopamine agonists 4.2%
Reported medication errors	74%	71%	89.9%	71.8%
Nil per mouth/unable to swallow orders	-	22%	-	65.6%
Unavailability of drugs	54%	12%	-	24.3%
Dopamine blockers administration	-	41%	21.3%	27.2%
Neurologist consulted	-	-	32.5%	24.5%

ER: emergency room.

The different methodological designs and clinical settings somewhat limit comparisons. However, the overall collective findings are relatively consistent. The first study included 35 PD patients who were admitted to the ER [22] and reported that 74% of had their PD medications stopped, doses omitted, or had medication that was prescribed inappropriately. The timing of medication doses was also observed to be a problem due to incorrect prescribing (68%), and unavailability of drugs (54%). Scheduling of nurses’ rounds (55%) also contributed to hospitalization issues. There was a reported “poor understanding” of contraindicated drugs in PD, and 11% of patients were prescribed dopamine blocking medications. The second study examined the pharmacological management of 51 PD patients who were assessed during elective surgical admissions [23]. This study reported missed dosages of PD medications in 71% of admissions. The reasons cited for missed or late dosages were “unable to swallow” in 14%, “out of stock” in 12%, and “nil per mouth” in 8%. Inappropriate antidopaminergic drugs were prescribed in 41% of admissions, with procholorperazine (34%) and cyclizine (12%) as the most common. The final study investigated discrepancies in medication administration during the hospitalization of 89 patients [24], and reported incorrect administration in 90% of patients with 8.3% of the total prescribed dosages omitted and 7.7% administered late. Contraindicated medications were prescribed in 21.3% of patients.

Our cohort is to date the largest that has examined dopaminergic medications errors in hospitalized patients. We observed that written orders for dopaminergic drugs (approximately 90% of cases) were more commonly prescribed in simple hospital-based schedules (BID, TID, or QID) rather than in specific time based schedules. Importantly, when dopaminergics were appropriately prescribed with time schedules, only 10% of levodopa and 8% of dopamine agonist dosages were not administrated within the expected hour or were omitted with nil per os orders, and non-availability was the most common reason for omission. Although we observed an overall rate of 30% of delayed or missed total levodopa dosages, this rate jumps to 50% when examined per hospital encounter. These numbers were comparable when analyzing dopamine agonist related errors. There were 71.8% of encounters with at least one delayed or missed levodopa dose and 54.1% with a missed dopamine agonist dose. It was alarming that contraindicated dopamine blocking agents were administered in 78.9% (71/90) of cases. Quetiapine was the most commonly administered non-contraindicated dopamine blocker (22/24). Quetiapine has been shown to affect motor symptoms, yet to a much lesser degree than other antipsychotics. Clozapine was hardly used in our cohort, and this may have been due to the required blood monitoring.

The most surprising finding was that though neurologists were consulted in less than 25% of the encounters, their involvement did not impact whether there were delayed or missed doses of levodopa, or whether a dopamine blocker was administered. This issue could have been due to a number of reasons, including a lack of awareness of frequent medication errors in the hospitalized PD patient, and that orders were frequently written by the primary team caring for the patient even when the specialist was consulted.

The retrospective nature of this study limits the direct attribution of extended lengths of stay in this cohort to the rate of PD medication errors. A possible inaccurate documentation of medication administration by the nursing department should be considered a potential unavoidable weakness. A selection bias should also be taken into account. Patients from a single center were used for the study, which may not represent the general population or practices might be unique to our institution. Confounders not included in the scoring system for comorbid conditions used were not included in the analysis and could be related to length of stay and medication dosing (such factors as non-motor PD symptoms). However, our careful control of demographic variables and comorbidities suggest that medication errors may be an important factor. There is a lack of indices of risk stratification to predict functional decline in the hospitalized PD patients, though it has been studied previously in the geriatric population [25]. Future studies using these tools could help in decision-making and also to predict outcomes. In addition, we observed a significant difference in mean length of stay in those patients admitted with a higher risk of ESI, which could be an important confounder for length of stay. Although a direct relationship between the wrong administration of dopaminergics and length of hospital stay could not be attributed based on the severity of admission risk, we should consider it a contributing factor to the prolonged length of stay. Whether addressing the issues uncovered by this study will lead to shortened lengths of stay for the hospitalized PD patient remains to be tested. A recent pilot study prospectively explored the effect of utilizing a specialized Parkinson’s disease care unit (SPDU). This study revealed that fewer medications were omitted, more medications were administered on time, and that patients had a shorter length of stay and a better overall experience [26]. The authors suggested the need for a larger randomized controlled study to confirm these findings, though admittedly this model would not likely be feasible on a larger scale. Though the need for guidelines has been addressed by a number of authors, no current guidelines exist for the management of the hospitalized PD patient, and there is an urgent need for better management protocols [27, 28].

## Conclusions

In summary, hospitalized PD patients are frequent victims of missed dosages of dopaminergic medications and administration of contraindicated medications, and these could be associated with prolonged length of stay. Efforts should be made to increase the awareness of the magnitude and impact of medication errors in PD has on prolongation of hospital stays.
